# Does Mutualism Drive the Invasion of Two Alien Species? The Case of *Solenopsis invicta* and *Phenacoccus solenopsis*

**DOI:** 10.1371/journal.pone.0041856

**Published:** 2012-07-23

**Authors:** Aiming Zhou, Yongyue Lu, Ling Zeng, Yijuan Xu, Guangwen Liang

**Affiliations:** Red Imported Fire Ant Research Center, South China Agricultural University, Guangzhou, China; Stanford University, United States of America

## Abstract

Although mutualism between ants and honeydew-producing hemipterans has been extensively recognized in ecosystem biology, however few attempts to test the hypothesis that mutualism between two alien species leads to the facilitation of the invasion process. To address this problem, we focus on the conditional mutualism between *S. invicta* and *P. solenopsis* by field investigations and indoor experiments. In the laboratory, ant colony growth increased significantly when ants had access to *P. solenopsis* and animal-based food. Honeydew produced by *P. solenopsis* also improved the survival of ant workers. In the field, colony density of *P. solenopsis* was significantly greater on plots with ants than on plots without ants. The number of mealybug mummies on plants without fire ants was almost three times that of plants with fire ants, indicating a strong effect of fire ants on mealybug survival. In addition, the presence of *S. invicta* successfully contributed to the spread of *P. solenopsis*. The quantity of honeydew consumption by *S. invicta* was significantly greater than that of a presumptive native ant, *Tapinoma melanocephalum*. When compared with the case without ant tending, mealybugs tended by ants matured earlier and their lifespan and reproduction increased. *T. melanocephalum* workers arrived at honeydew more quickly than S. invicta workers, while the number of foraging *S. invicta* workers on plants steadily increased, eventually exceeding that number of *T. melanocephalum* foragers. Overall, these results suggest that the conditional mutualism between *S. invicta* and *P. solenopsis* facilitates population growth and fitness of both species. *S. invicta* tends to acquire much more honeydew and drive away native ants, promoting their predominance. These results suggest that the higher foraging tempo of *S. invicta* may provide more effective protection of *P. solenopsis* than native ants. Thus mutualism between these two alien species may facilitate the invasion success of both species.

## Introduction

Mutualistic interactions occur commonly between hemipteran and invasive ants, which are notorious for their aggressiveness and high colony density [1]–[3]. Ecosystem domination by invasive ants is effectively strengthened by the collection and exploitation of honeydew and plant extrafloral nectar [2]–[5]. Colony growth of both insects is facilitated by the interactions between ants and honeydew-producing hemipterans [6]. The growth of honeydew-producing hemipterans has been shown to be facilitated by ant tending [2], [7]–[10]. Increases in hemipteran density attract more aggressive and dominant ants, however, resulting in the dislodging of native ants in many cases [11], [12].

As one of the most important threats to ecosystems, many invasive species are recognized by their extreme aggression and broad omnivory [2], [13]–[15]. Native communities can be significantly damaged by invasive species [2]. Ecosystems are commonly disrupted by invasive species that exploit existing mutualisms [16]. Hemipteran communities and colonies can be supported by ant tending because certain ant species protect honeydew-excreting hemipterans from their natural enemies [17]. For example, the density of the obscure mealybug, *Pseudococcus viburni,* in California coastal vineyards significantly increased when tended by the Argentine ant, *Linepithema humile,* and the density of encyrtidae parasitoids and predators decreased in vineyards with the Argentine ant [18]. Besides reducing the mortality risk of fungal infection[9], [19], [20], ant tending may enhance aphid colonies by the stimulation of feeding and honeydew excretion [21], [22]. Mutualism between ants and aphids may have positive effects on the aphid life cycle. For example, individual *Metopeurum fuscoviride* Stroyan aphids tended by *Lasius niger* ants lived longer, matured earlier, had a higher rate of reproduction, and had a higher expected number of offspring than aphids that were not tended by ants [23].

In exchange for tending of the hemipterans, the ants receive large amounts of honeydew in such a consumer-resource mutualism[24]. Tended hemipterans usually supply abundant honeydew, which is considered to be an important food resource for ants because it contains sugars mixed with various amino acids and energy-rich materials [25]–[28]. Invasive ant species are usually omnivorous and have enormous populations; they not only utilize animal-based food resources in their surroundings but also feed on plant juices and the honeydew excreted by hemipteran insects, which can facilitate colony growth to some extent [2], [3], [29].

Compared with plant-based food resources such as plant juices and honeydew, the effects of animal-based food resources on the population growth of omnivorous ants are much more significant [30]. Animal tissues are rich in protein, which can significantly facilitate the growth and development of ant larvae [31], [32]. However, when animal-based food is scarce, honeydew becomes indispensable for sustaining many ants. Other research has indicated that laboratory colonies of *S. invicta* are substantially enhanced when supplied with honey water [33]. Colonies of *S. invicta* reared in the absence of other insects ceased brood production entirely, and colony growth was reduced by 60% because of the lack of sugar-water supply [34]. Ant workers cannot digest solid food directly, and honeydew and plant juices are composed mainly of carbohydrates that can supply energy for worker activity [29], [35], [36].

**Figure 1:**
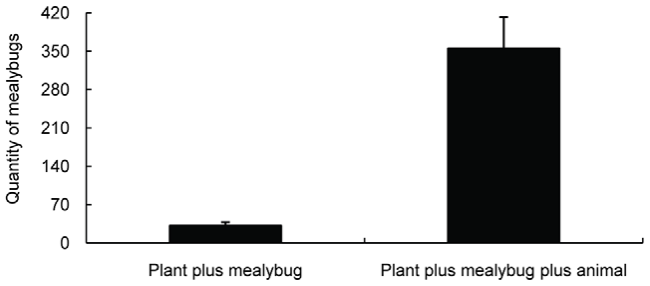
Effect of tending by *S. invicta* on colony growth of mealybugs when provided with different foods (average ± SE). The treatments differed significantly ( independent *t*-test, *P*>0.05).

The red imported fire ant, *Solenopsis invicta,* is native to South America and is a dangerous invasive species in the United States [37]. Many studies have reported important ecological effects of *S. invicta,* including decreased local biodiversity and the disruption of native ant communities [37], [38]. Similarly, negative effects of *S. invicta* on agriculture and forestry production, human health and poultry production have been recorded in South China [39]. The mealybug *Phenacoccus solenopsis* Tinsley (Hemiptera: Pseudococcidae) is native to the US and has spread throughout the world [40]. It has a wide geographic distribution and can be found in Central America, South America and Africa [41], [42]. It has been found to cause serious damage to cotton crops in India and Pakistan in 2005 and to *Hibiscus rosa-sinensis* in Nigeria [43], [44]. Recently, *P. solenopsis* was reported to be an important invasive species in South China [45]. Obviously, it is a common ecological phenomenon for the mealybug, *P. solenopsis* to form close relationships with ants, similar to that between aphids and ants. Few studies, however, have investigated such a relationship.

The mechanisms promoting invasion is a core issue in invasion biology, and the study of the role of the interaction between species in the invasion process, including competition and facilitation, has become heated in recent years. Liu et al. [46] explored the asymmetric mating mechanism of whitefly invasion from the point of view of species competition. Meanwhile, the effect of mutualism and positive facilitation on successful invasion has been commonly acknowledged [47]–[49]. For example, some studies have found that the invasion of some species may promote other species' successful settlement and invasion by changing the habitat characteristics and species composition of the infested area [47]. The interaction between invasive and native species [50], and the facilitation of the invasion process by the interaction between an alien insect and its symbiotic bacteria [51] have been documented. However, few studies have addressed the mutualism and its contribution to invasion of two alien insect species.

**Table 1:** The effect of food supply on colony growth in *S. invicta*.

Treatments	Mean measured weight (g)
no food	0.267±0.0547c
plant	0.298±0.014c
plant plus mealybugs	0.495±0.1347c
animal	10.413±1.7829b
plant plus animal	11.623±1.8143ab
plant plus mealybugs and animal	18.029±3.0780a

Data in a given column followed by the same letter are not significantly different from each other (*P>*0.05, Mann-Whitney test).

Previous studies have suggested that the invasive ant *S. invicta* tends the invasive mealybug *Antonina graminis* (Maskell) extensively and actively constructs shelters around these insects. Additionally, honeydew produced by *A. graminis* is an important component of the diet of *S. invicta* [3]. Helms and Vinson [52] indicated that colonies of *S. invicta* grew substantially larger when supplied with insect prey and honeydew produced by *A. graminis* than those in other treatments with access to unlimited insect prey. This study also showed that nutritional resources for *S. invicta* were unlikely to be acquired directly from hemipteran host plants; rather, they were provided indirectly from honeydew. In a previous field investigation, we found that fire ant workers foraged more frequently on plants when mealybugs were present [53], suggesting that *S. inivcta* in south China may be deriving benefits from another invasive insect, *P. solenopsis.* Based on these results, we developed the following hypothesis: Compared with native ants, fire ants are able to form closer mutually-beneficial relationships with *P. solenopsis,* which may enhance colony development and facilitate their invasion success. To test this hypothesis, we conducted a series of experiments to examine the effects of honeydew excreted by *P. solenopsis* on the growth of *S. invicta* colonies. We also determined the effects of fire ant tending on the growth of colonies of *P. solenopsis* in the field. Furthermore, the impacts of ant tending by *S. invicta* and native ants on single individuals and growth of small colonies of *P. solenopsis* were examined. The results of these experiments may provide insights into how mutualism between invasive ants and exotic honeydew-producing hemipterans can promote the invasion success of both partners.

**Figure 2:**
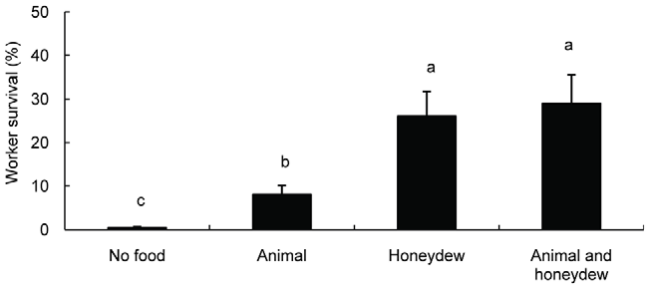
Effect of food supply on worker survival of *S. invicta*. Each bar represents the mean of four treatments (average ± SE). Bars labeled with the same letter are not significantly different from each other (P>0.05, LSD).

## Materials and Methods

### Plants

*Hibiscus rosa-sinensis*, a Chinese native species cultivated worldwide, was purchased from a commercial horticultural farm. Each plant was approximately 25–30 cm in height and had 25–30 true leaves. All plants were cultivated in plastic flowerpots (the diameters of the upper and lower edges were 18 cm and 14 cm, respectively, with a height of 17 cm) in greenhouses.

**Figure 3:**
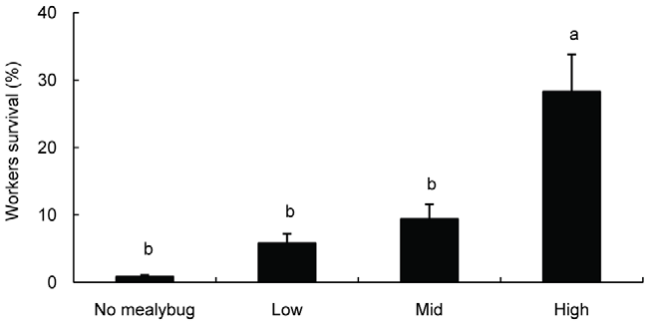
The effect of mealybug density on worker survival in *S. invicta*. Each bar represents the mean (±SE) of the following four treatments: (1) nomealybugs; (2) a low mealybug density of 30 per plant; (3) a medium mealybug density of 60 per plant; and (4) a high mealybug density of 120 per plant. Bars labeled with the same letter are not significantly different from each other (P>0.05, LSD).

### Insects

Colonies of *P. solenopsis* were collected from the campus of South China Agricultural University and fed on *H. rosa-sinensis*. The 1^st^ instar mealybug nymphs were inoculated on each plant and raised for several generations. *H. rosa-sinensis* plants with established mealybug colonies were used for subsequent experiments. All colonies were reared in the laboratory with the temperature maintained at 27±2°C and a relative humidity of 60–70%.

**Figure 4:**
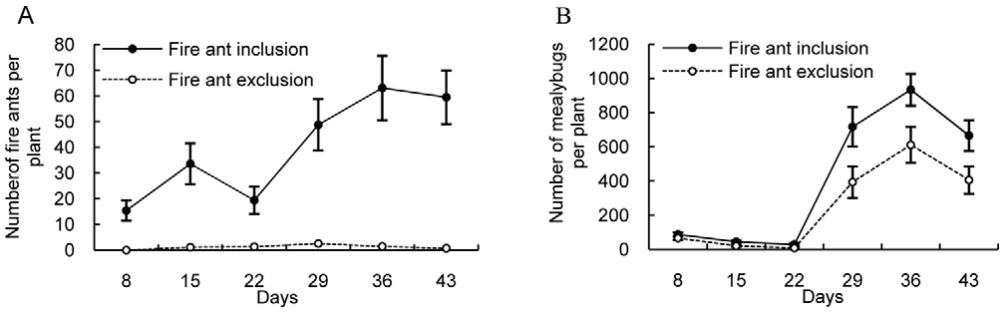
Mean number (±SE) of *S. invicta* per plant (A) and effect of ant tending by *S. invicta* on the density of mealybug colonies (B) in fire ant-tended plots (•) and fire ant-excluded plots (○).

**Figure 5:**
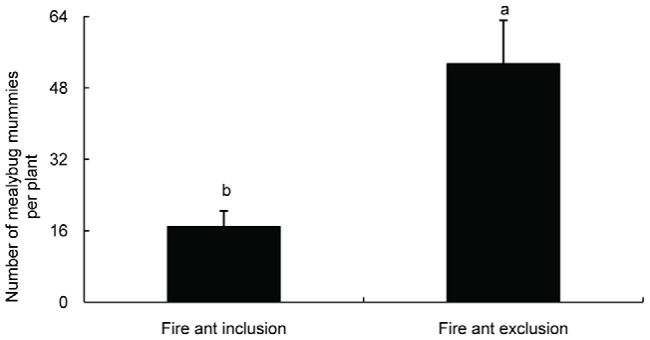
Effect of *S. invicta* tending on the number of mealybug mummies (average ± SE). Lower numbers of mummies in the presence of fire ants indicated that fire ants drive parasitic wasps away from the mealybugs. Bars labeled with the same letter are not significantly different from each other (*P*>.05, independent *t*-test).

We collected 16colonies of *Tapinoma melanocephalum*, a highly competitive and sugar-feeding species [54] from the campus of South China Agricultural University. This species is a worldwide invader whose native range is unknown but is believed to come from Africa or Asia [55]. It has been in southern China for a long time where it is fully established as a resident species. For the purposes of this paper it is considered a presumptive native of southern China.

**Figure 6:**
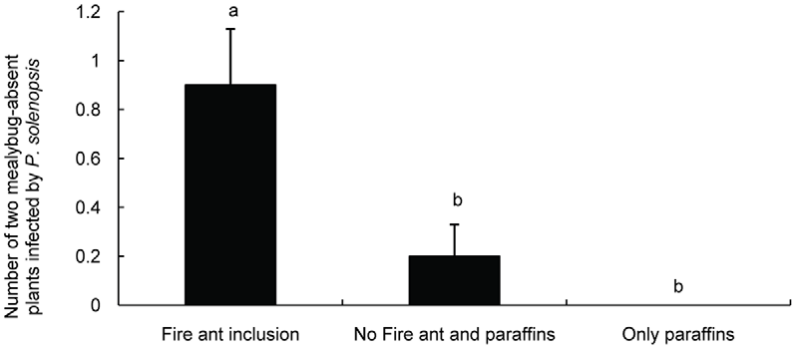
Effect of ant tending by *S. invicta* and paraffins on the spread of *P. solenopsis* (average ± SE). Bars labeled with the same letter are not significantly different from each other (*P*>0.05, Mann-Whitney test).

A total of 51 newly established colonies of *S. invicta* were collected from a suburb of Guangzhou and reared in plastic boxes (116 L). All colonies were separated from the soil by dripping water into plastic boxes until the colonies floated (Jouvenaz et al. 1977). Each colony was divided into more than 20 sub-colonies (approximately 1.0 g) using a microbalance (Sartorius, BS, 224S). Each sub-colony included one queen and adult workers, pupae, larvae, and eggs. The ants were placed in a 9-cm plastic Petri dish, which served as an artificial nest. *T. melanocephalum* sub-colonies were maintained with tubes filled with distilled water plus 10% honey solution. Fire ants were given fresh live *Tenebrio molitor* worms, frozen crickets, and a 10% solution of honey mixed with water (50 ml) weekly.

**Figure 7:**
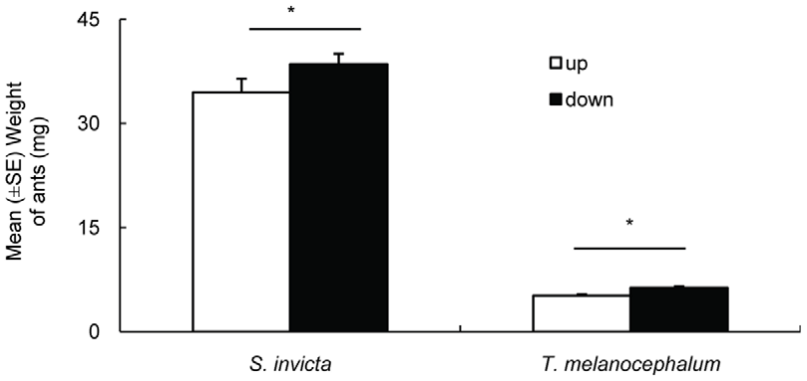
Difference in worker weights before and after foraging (average ± SE). * on the two bars indicate significantly different from each other (*P*>0.05, paired t-test).

**Figure 8:**
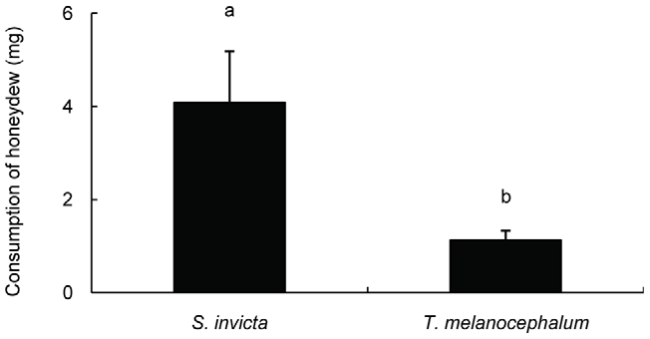
Difference in honeydew consumption between *S. invicta* and *T. melanocephalum* (average ± SE). Bars labeled with the same letter are not significantly different from each other (*P>*0.05, Mann-Whitney test).

For each experiment, we used a number of different colonies. Each colony was divided into a number of sub-colonies, with only one sub-colony per colony used for each treatment. Thus, each colony was represented by only one sub-colony per treatment in each of the experiments.

### Experimental design

#### Experiment 1: Effect of food composition on colony growth of *S. invicta*

We placed a small fluon-coated plastic case (40 cm×28 cm×22 cm) that was loaded with soil into a fluon-coated plastic box (52 cm×39 cm×30 cm). Each small plastic case was seeded with a colony of *S. invicta*. By 24 h, the ants had constructed a new nest in the soil. One hundred 1^st^ instar mealybugs were transferred to potted plants. A plastic hose was used to build a bridge between the ant nest and the base of the plant stem for worker foraging. Pots were coated with fluon to prevent ant escape. Our experiments included the following treatment groups: (1) water supply only; (2) one potted *H. rosa-sinensis* plant placed in the plastic drum; (3) live worms plus frozen cockroaches; (4) one potted *H. rosa-sinensis* plant plus live worms and cockroaches; (5) one *H. rosa-sinensis* plant infected with *P. solenopsis*; and (6) one *H. rosa-sinensis* plant infected with *P. solenopsis* plus live worms and cockroaches. One test tube (15×1.5 cm) filled with distilled water and sealed with a cotton plug was placed into a small plastic case as a constant source of water. To prevent drying, the soil in each small plastic case received sprayed water every 3 days. Each treatment was replicated 15 times. The experiments lasted for 8 weeks after which time, all surviving ants were extracted, counted, weighed, and measured using a microbalance (Sartorius, BS, 224S).

**Figure 9:**
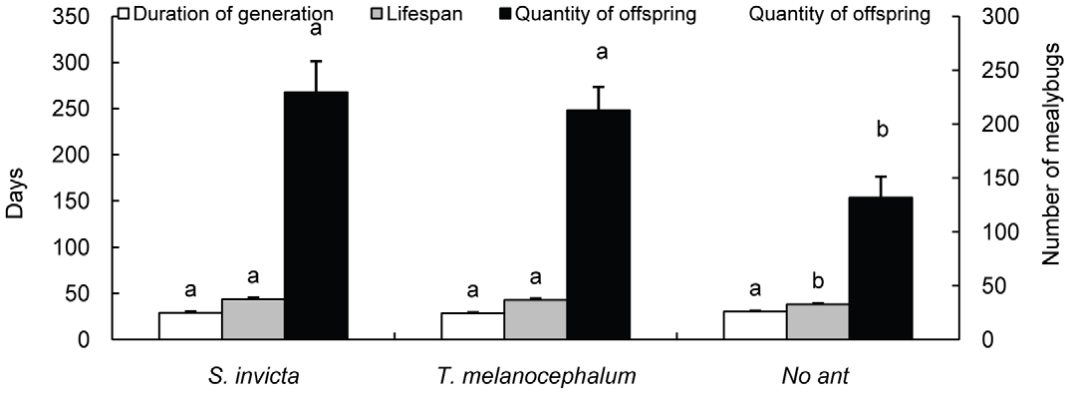
Effect of ant tending on the length of the developmental period, lifespan and fecundity of single mealybugs (average ± SE). Bars of the same color labeled with the same letter are not significantly different from each other (*P>*0.05, LSD).

#### Experiment 2: The effect of food variety on the survival of worker ants

One gram (approximately 950–1000 individuals) of workers was placed in a plastic box (26 cm×18 cm×8 cm) with distilled water supplied every day, and a petri dish (7 cm diameter) with moist plaster was used as an artificial nest. One hundred 1^st^ instar mealybugs were transferred to the potted plants. Paraffin wax was used to coat the base of the plants to prevent escape by the mealybugs. We placed the potted plant and artificial ant nest in a large plastic box (50 cm×40 cm×16 cm) after the mealybugs had colonized the plants (reared previously). At the beginning of the bioassay, a plastic hose was used to build a bridge between the ants' nest and plant seedlings to allow worker foraging. The ants were raised with access to different food supplies: (1) distilled water only; (2) live worms; (3) a potted plant inoculated with *P. solenopsis*; (4) live worms and a potted plant inoculated with *P. solenopsis*. The experiments were conducted for 5 weeks. We counted the number and measured the weights of the surviving ants using a microbalance (Sartorius, BS, 224S). Each food supply treatment was replicated 10 times. We calculated the survival rate of the ants as 100% × the number of surviving ants after tests/the number of surviving ants before tests.

**Figure 10:**
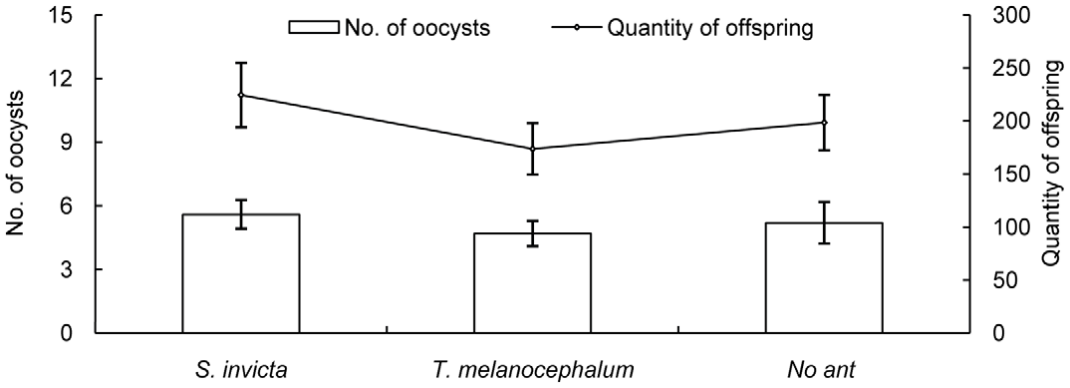
The effect of ant tending on the fecundity and the quantity of oocysts produced by small colonies of mealybugs (average ± SE).

#### Experiment 3: Effect of mealybug density on the survival of worker ants

This experiment involved four treatments with different densities of mealybugs on *H. rosa-sinensis*. First, 1^st^ instar nymph mealybugs were transferred into potted *H. rosa-sinensis* plants. The treatments were as follows: (1) no mealybugs; (2) a low mealybug density of 30 per plant; (3) a medium mealybug density of 60 per plant; and (4) a high mealybug density of 120 per plant. We determined the effects of mealybug density on the survival of the ant workers. The experimental protocols were performed as described above, with 10 replicates per condition. The tests were finished after 5 weeks. The number of surviving ants was counted. Worker survival rate was calculated as 100% × the number of surviving ants after the tests/the number of surviving ants before the tests.

**Figure 11:**
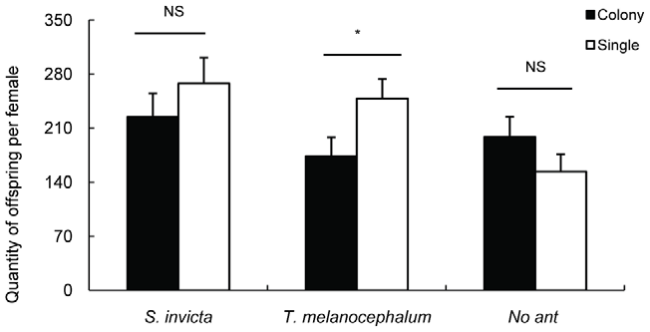
The effects of ant tending on the fecundity of individuals and small colonies of mealybugs (average ± SE). * and NS on the two bars indicate significantly (*P*<0.05) and not significant (*P*>0.05) different from each other, respectively according to an independent *t*-test.

#### Experiment 4: Effect of ant tending by *S. invicta* on colony growth of *P. solenopsis* in the field

This investigation was conducted in the fields of South China Agricultural University. Plots were prepared for growing *H. rosa-sinensis*. The area of each plot was approximately 25 m^2^ (5 m×5 m). Our experiments were conducted as follows: (1) plots were supplied with colonies of *S. invicta*; (2) plots without fire ant infestation were chosen to exclude colonies of *S. invicta*, and the base of the main stem of the plants was covered with paraffin, (3) plots without fire ant infestation were chosen to exclude colonies of *S. invicta*, but the base of the plant stem was not covered with paraffin. Sizable colonies of *S. invicta* were inoculated on the appointed plots. We then checked whether the transferred colonies had survived, and a second colony of *S. invicta* was provided if the first colony had not successfully established. In each treatment, a circle with its center point at the ant colony and a radius of approximately 1 m was drawn, and, four *H. rosa-sinensis* plants were placed within the circle at 90-degree angles. Two neighboring plants had been infected with mealybugs, whereas the other two (also neighboring) plants were uninfected. Individual 1^st^ instar mealybug larvae were transferred onto the plant via small plastic tubes with cotton plugs prior to their introduction to the study site. For this transfer, we placed four tubes, each containing 100 individuals, on the top branches of each *H. rosa-sinensis* plant. When the plug was removed, the nymphs crawled out from the tubes and began sucking the tender plant leaves. We investigated the hypothesis that the presence of *S. invicta* colonies facilitates the growth and spread of *P. solenopsis* colonies. One mealybug-infected plant was selected randomly for our investigation (a different plant was observed if the colony of *P. solenopsis* had disappeared). The number of foraging workers moving up or down the plant trunk during a 3-min period was counted, and the density of live *P. solenopsis* was determined every 7 days. The quantity of mealybug mummies and the probability of *P. solenopsis* spread were also measured at the end of the investigation. We assumed that the probability of spreading was 100% if both mealybug-absent plants were colonized by *P. solenopsis*, 50% if one was infected and 0 if neither plant was infected. Each treatment was replicated 10 times.

**Figure 12:**
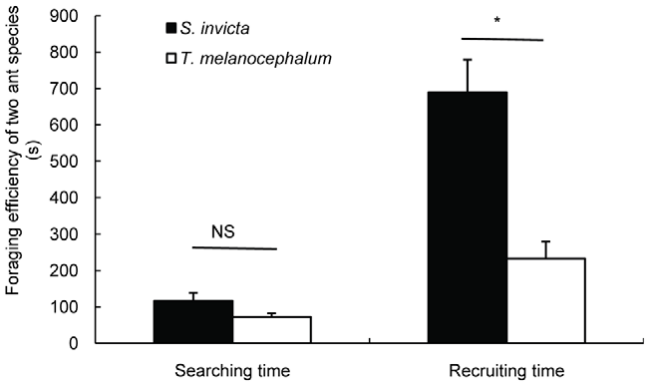
Different foraging intensity between *S. invicta* and *T. Melanocephalum* (average ± SE). * and NS on the two bars indicate significantly (*P*<0.05) and not significantly (*P*>0.05) different from each other, respectively according to an independent *t*-test.

#### Experiment 5: Consumption of honeydew by *S. invicta* and *T. melanocephalum*

*H. rosa-sinensis* seedling leaves were inoculated with 60 3^rd^ instar mealybugs, which were subsequently reared on the plants. Artificial nests (one queen and 1 g workers) of *S. invicta* and *T. melanocephalum* were transferred to individual plastic cases (40 cm×28 cm×22 cm). The workers began to build the nests immediately. After 24 h, mealybug-infected plants were placed into each plastic case. A plastic hose was used to build a bridge between the ants' nest and the stem of the plant to allow worker foraging. We collected 30 random workers from the bottom stalk at the beginning of the experiment, as they were moving toward the mealybugs, and 30 more were collected after 24 h, as they were returning from the mealybugs. The weight of the ants collected before and after foraging was measured by a microbalance (Sartorius, BS, 224S). Every treatment was replicated 10 times.

**Figure 13:**
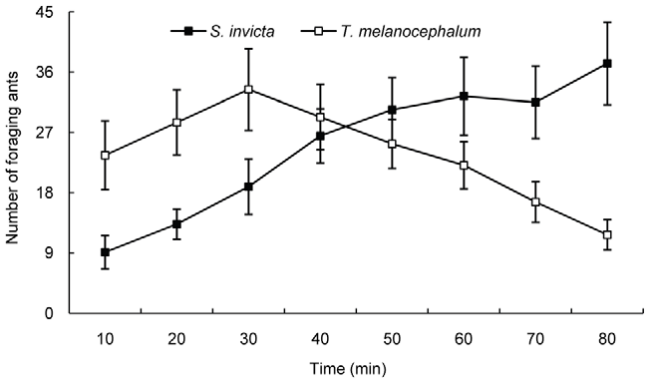
Comparison of the foraging dynamics of *S. invicta* and *T. Melanocephalum* workers on *H. rosa-sinensis* plants (average ± SE).

#### Experiment 6: Effect of tending by *S. invicta* and *T. melanocephalum* on single *P. solenopsis* mealybugs

Single 1^st^ instar mealybugs were transported to a tender leaf of a potted plant and reared for 24 h. At the same time, artificial nests of *S. invicta* and *T. melanocephalum* were transferred to individual plastic cases. Workers began to build nests immediately. After 24 h, the mealybug-infected plants were placed into the plastic cases. A plastic hose was used to build a bridge between the ants' nests and the bottom of the plant to allow worker foraging. We checked each live mealybug daily and recorded its survival and number of offspring. All offspring were removed from the plant. We computed the developmental duration period (age at first reproduction), lifespan (age at the end of reproduction) and fecundity (number of offspring produced by each individual). Parallel experiments were conducted in ant-excluded plants. Each treatment was replicated 25 times. Observations continued until the last mealybug died.

#### Experiment 7: Effect of tending by *S. invicta* and *T. melanocephalum*on small colonies of *P. solenopsis*

Forty 1^st^ instar mealybugs were transported to the tender leaves of potted plants and reared for 24 h. At the same time, the artificial nests of *S. invicta* and *T. melanocephalum* were transferred to individual plastic cases. After 24 h, mealybug-infected plants were placed into the plastic cases. A plastic hose was used to build a bridge between the ants' nests and the bottom of the plant to allow worker foraging. We checked each plant daily and recorded the survival and number of offspring of the mealybugs. All offspring were carefully removed from the plant. Parallel experiments were conducted in ant-excluded plants. Each treatment was replicated 10 times. The investigation lasted approximately 8 weeks.

#### Experiment 8: Competition for honeydew between *S. invicta* and *T. melanocephalum*

Each colony (one gram workers and one queen) of *S. invicta* and *T. melanocephalum* was transferred to a plastic box (26 cm×18 cm×8 cm). One hundred 1^st^ instar mealybugs were transferred to *H. rosa-sinensis* plants. After abundant honeydew has present on plants, each nest of *S. invicta* and *T. melanocephalum* received a plastic pipe (with the length and the diameter of 35cm and 0.8cm, respectively) which was used to build a bridge between the nest and plant stem for worker foraging. In order to contain the ants and mealybugs, the base of the plant stems were coated with ceresin wax. A colony of *S. invicta* and *T. melanocephalum* was connected with the plants. We recorded the search time (time for the first ant to arrive at the honeydew) and recruitment time (time for 10workers to be present on the plant) of *S. invicta* and *T. melanocephalum*. In addition, to determine the level of competition between *S. invicta* and *T. melanocephalum* for honeydew, we connected two ant colonies to one plant. We counted the number of foraging workers of the two ant species on the plant every 10minutes. The experiment lasted for 80 minutes. All treatments were replicated 10 times.

We conducted the field studies in areas where fire ants and mealybugs occur in the field and no specific permits were required for the described field studies. The land used in the study area is not privately-owned or protected in any way and the field studies did not involve endangered or protected species.

### Statistical analysis

To compare the differences in colony weight and survival rate between treatments, survival rate between densities, quantity of foraging ant workers per plant and probability of *P. solenopsis* spread between ant-including and ant-excluding plots, and the developmental duration, lifespan, searching time and recruitment time of workers, all data were tested for normal distribution by Shapiro-Wilk test and for homogeneity of variances by Levene's test. If the data were normally distributed and had similar variances, then one-way analysis of variance (ANOVA) using Type III sum of squares was performed to compare means among all measured variables. When ANOVA results were significant, multiple comparisons of means were performed with Tukey HSD post-hoc analysis. If the data did not have similar variances, the non-parametric Kruskal-Wallis test for comparing the median was applied and also the Mann-Whitney test (or the two-sample Kolmogorov-Smirnov test) for multiple comparisons among the different groups if the results of the Kruskal-Wallis test showed significant differences at the 0.05 significance level.

The density of *P. solenopsis* per plant, quantity of mealybug mummies on plants in ant-including and ant-excluding plots, changes in ant weight and honeydew consumption between the two ant species, and differences in reproductive quantity among single mealybugs and small colonies of *P. solenopsis* tended by *S. invicta* and *T. melanocephalum* were analyzed with independent-samples t-tests. We used paired tests to compare worker weights before and after foraging.

In addition, all proportion data, such as proportion spread or survival, were binomially distributed and analyzed after arcsin square root transformation. All statistical analyses were conducted using SPSS version 14.0 (SPSS Inc., Chicago, IL, USA).

## Results

### 1 The effect of food composition on colony growth in *S. invicta*

The variable food composition resulted in markedly different colony masses under different treatments (χ2 = 65.187, p<0.001). Colony mass was not obviously different between the animal food only treatment and the animal food with plants treatment (Mann-Whitney test, U = 97.00, P = 0.520). However, colony mass in the treatment provided with animal food with plants and mealybugs was significantly greater than that receiving only animal food (Mann-Whitney test, U = 64.00, P = 0.044, Table 1). In addition, colony growth of mealybugs was greater when fire ants were provided with animal source of foods than without (t = −5.629, P<0.001, Fig. 1).

### 2 The effect of food variety on survival of worker ants in *S. invicta*

The survival of *S. invicta* workers differed in different food varieties (F = 17.841, *P*<0.001, Fig. 2). Worker ants provided with distilled water only had the lowest survival. There was a significant difference between the water-only and animal-based food conditions (*P* = 0.01). The survival of workers was significantly increased when worker ants had access to mealybugs or mealybugs plus animal-based food compared with the other treatments (*P*<0.01, Fig. 2); there was no difference, however, in the survival between these two treatments (*P* = 0.638).

### 3 The effect of *P. solenopsis* density on ant worker survival in *S. invicta*

The survival of workers increased significantly with greater mealybug density (F = 21.645, df = 3, *P*<0.001, Fig. 3).

### 4 Foraging dynamics of *S. invicta* workers and mealybug density on plants

The average fire ant forging activity was markedly higher in the ant-tended plants than in the ant-excluded plants (t = −4.756, df = 5, P = 0.005, Fig. 4a). Few workers were present on ant-excluded plants (Fig. 4a). There was no obvious difference in mealybug density between the ant-tended and ant-excluded plants in the first observation (Day 8) (t = −1.328, df = 18, P = 0.201, Fig. 4b), whereas in all of the later observations, there was a significant difference between ant-tended and ant-excluded plants (t = −2.202, df = 18, P = 0.041; t = −2.503, df = 10.344, P = 0.031; t = −2.196, df = 18, P = 0.041; t = −2.297, df = 18, P = 0.034; t = −2.161, df = 18, P = 0.044, respectively, Fig. 5).

### 5 Effect of ant tending by *S. invicta* on the quantity of mealybug mummies

Our results indicate that the number of mealybug mummies on ant-excluded plants was significantly greater than that on ant-tended plants (Mann-Whitney test, U = 13.5, *P* = 0.006, Fig. 5), which indicated that fire ants drive parasitic wasps away from the mealybugs. The probability of *P. solenopsis* spread was conspicuously different among the three treatments (F = 9.277, df = 2, *P* = 0.001, Fig. 6). There was no significant difference in the probability of *P. solenopsis* spread between the treatments of plants with and without paraffin in ant-excluded plots (Mann-Whitney test, U = 40.0, *P* = 0.146).

### 6 Honeydew consumption by *S. invicta* and *T. melanocephalum*

We recorded the difference in ant worker weights between ants moving in two different directions (i.e., prior to honeydew consumption and after honeydew consumption) in the two species. Paired t-tests showed that returning workers were significantly heavier than outgoing workers on plants with *P. solenopsis* for both ant species (*S. invicta*: t = −3.713, df = 9, *P* = 0.005; *T. melanocephalum*: t = −5.546, df = 9, *P*<0.001; Fig. 7). Therefore, both ant species were able to collect abundant honeydew from *P. solenopsis*-infested plants. However, we detected a significantly greater amount of honeydew consumed by workers *S. invicta* than by workers of *T. melanocephalum* (Mann-Whitney test, U = 9.0, *P* = 0.002; Fig. 8).

### 7 Effect of *S. invicta* and *T. melanocephalum* tending on a single *P. solenopsis*

Single mealybugs tended by *S. invicta* and *T. melanocephalum* matured an average of 1.2 and 1.7 days earlier than mealybugs tended by no ants, respectively, but there were no significant differences among the three tending treatments (F = 0.736, df = 2, *P* = 0.489; Fig. 9). The lifespan and reproduction of single mealybugs were significantly increased by tending by both ant species. Tending by *S. invicta* and *T. melanocephalum* extended the lifespan of single mealybugs by 5.6 d and 4.7 d, respectively (F = 3.487, df = 2, *P* = 0.046; Fig. 9), and the number of offspring increased by 114.2 and 94.7 individuals, respectively (F = 5.190, df = 2, *P* = 0.013; Fig. 9).

### 8 The effect of *S. invicta* and *T. melanocephalum* tending on small colonies of *P. solenopsis*

There was no obvious difference in the reproduction of colonies of *P. solenopsis* in the presence and absence of ant tending (F = 0.880, df = 2 *P* = 0.426, Fig. 10). The number of offspring and oocysts produced by *P. solenopsis* colonies tended by *S. invicta* and *T. melanocephalum* were also similar (F = 0.347, df = 2 *P* = 0.710, Fig. 10).

### 9 Offspring production in single and small colony *P. solenopsis* under tending by *S. invicta* and *T. melanocephalum*

An independent-samples t-test showed that reproduction of *P. solenopsis* colonies decreased significantly when tended by *T. melanocephalum* (t = 2.127, df = 19, *P* = 0.047, Fig. 11). In contrast, there was no significant difference in the reproduction of colonies on single *P. solenopsis* when tended by *S. invicta* (t = 0.958, df = 16, *P* = 0.353, Fig. 11). Without ant tending, there was no distinct difference in reproduction between colonies and single *P. olenopsis* (t = -1.298, df = 18, *P* = 0.211, Fig. 11).

### 10 Comparison of foraging behavior between *S. invicta* and *T. melanocephalum* to honeydew

Workers of *T. melanocephalum* arrived at honeydew more quickly than workers of *S. invicta*. But there was no significantly difference in searching time between the two ant species (t = 1.516, df = 17, *p* = 0.148, Fig. 12). Recruiting time of *T. melanocephalum* was significantly shorter than that of *S. invicta* (t = 4.520, df = 13.53, *p* = 0.001, Fig. 12).

Our results indicated that number of foraging workers of *T. melanocephalum* on plants increased continually in the first three investigations and decreased gradually after 30 minutes. The number of foraging workers of *S. invicta* on plants continuously increased during the entire observation period. Number of foraging workers of *T. melanocephalum* on plants was significantly more than the number of foraging workers of *S. invicta* in the first and second observation (t = −2.537, df = 12.94, *p* = 0.025; t = −2.843, df = 12.74, *p* = 0.014; Fig. 13). There was no significant difference in the number of foraging workers between the two ant species in the third, fourth, fifth and sixth observation (t = −1.966, df = 18, *p* = 0.065; t = −0.442, df = 18, *p* = 0.664; t = 0.848, df = 18, *p* = 0.408; t = 1.522, df = 18, *p* = 0.145; Fig. 13). However, by the seventh and eighth observation period, the number of foraging workers of *S. invicta* was significantly greater than that of *T. melanocephalum* (t = 2.76, df = 14.16, *p* = 0.015; t = 3.901, df = 11.38, *p* = 0.002; Fig. 13).

## Discussion

The fire ant *S. invicta* and the mealybug *P. solenopsis* are two invasive species that have vast populations in South China. *H. rosa-sinensis* is popularly cultivated in the parks and greenbelts where *S. invicta* frequently occurs, therefore it is likely that *S. invicta* encounters *P. solenopsis* in the field and that the two species establish a mutualistic relationship. Our study characterized the conditional mutualism between *S. invicta* and *P. Solenopsis* to determine whether these interactions may have facilitated the invasion of these two alien species.

Our results demonstrate that *S. invicta* benefits from the conditional mutualism in our experimental ecosystem. We found that an animal-based food supply could facilitate the growth of *S. invicta*. Previous studies also demonstrated that animal tissues were essential for ant colony growth [29], [30], [35]. Ant colonies supplied with live worms and cockroaches as prey and honeydew produced by *P. solenopsis* exhibited an obviously greater live mass than ant colonies supplied with animal-based food only, which suggests that the honeydew produced by *P. solenopsis* enhances the growth of *S. invicta* when insect prey is sufficient. However, the ant colonies lost most of their live mass when they were fed on honeydew only. We also found that access to honeydew strongly promotes worker ant survival. In contrast, the survival of the worker ant barely increased when supplied with animal-based food (Fig. 2). This may be explained by the fact that workers rarely ingest solid food resources [27], [36], [56]. Consistent with these observations, we also found that a high mealybug density significantly facilitated worker survival (Fig. 3). This result provides further evidence that carbohydrate-rich honeydew plays an important role in worker ant activity [28], [29], [35].

Such a consumer-resource mutualism not only influences the growth of fire ant colonies but also alters the density of *P. solenopsis* on *H. rosa-sinensis* plants. Mealybug survival was greater in treatments where ants were provided with animal-based food than in those without animal-based food (Fig. 1). This result suggests that *P. solenopsis* colony growth resulting from tending by *S. invicta* depends on the food supply of *S. invicta*. One reasonable conclusion based on this finding is that the absence of animal-based food may compel fire ants to exploit honeydew excessively, which may lead to a significant decrease in the fitness of *P. solenopsis* colonies on host plants. Our hypothesis is supported by a previous report indicating that ant tending has negative effects on developmental rate, growth rate and offspring production of aphid [57]. It seems that the mutualism between fire ants and mealybugs is dependent on the food supply and ant tending level. The variables underlying the interactions between these two invasive species require further study.

Our results demonstrate that tending by *S. invicta* ants could increase the density of *P. solenopsis* on *H. rosa-sinensis* plants by interfering with predation and parasitism by natural enemies (Fig. 5). We found that mealybug nymphs on ant-excluded plants were frequently preyed upon by the lady beetle *Menochilus sexmaculata* (Coleoptera: Coccinellidae) and infected by two parasitic species, *A. bambawalei* and *Acerophagus coccois* Smith. In contrast, the density of mealybug mummies was significantly lower and lady beetles were present less frequently on ant-tended plants, probably because fire ants attack visiting enemies on host plants. Similarly, cotton aphid populations and the predation of sentinel bollworm eggs were greater in the presence of *S. invicta* than in its absence [58]–[60]. Mealybug population growth was apparently facilitated by Argentine ants in California vineyards [61]–[63]. There were few mealybugs present on *H. rosa-sinensis* without ant tending and paraffin treatment, whereas more mealybugs were obviously present on ant-tended plants (Fig. 6). The elimination of the paraffins on the plants did not promote the appearance of *P. solenopsis*, which suggests that ant-tending by *S. invicta* may have positive effects on the short-range spread of mealybugs. We found that the ants could transport the unserviceable mealybugs (i.e., those that produced little honeydew) to their nests. However, few young mealybug nymphs, such as 1^st^ and 2^nd^ instar nymphs, were transferred directly by *S. invicta*. In fact, young mealybugs crawled more actively on plants than the adults. We observed that 3^rd^ instar nymphs and adults rarely moved to colonize a favorable location, which indicates that 1^st^ instar mealybugs have a higher rate of dispersal than other instar nymphs and adults. We inferred that the adults were removed by fire ants indiscriminately, which could increase the risk of spreading *P. solenopsis* because many unserviceable adults were reproductively active (i.e., contained offspring in their oocysts), even though they did not produce much honeydew. In the course of removal, 1^st^ instar nymphs were highly capable of invading other plants. It has been shown that foraging ants of *Lasius niger* commonly removed dead aphids from the colony under laboratory conditions [23]. These results support the conclusion that ants can recognize low-value mealybugs and remove them.

Resource competition has been considered one of the key factors shaping the reduction of native ant diversity by *S. invicta* [37]. Our result confirms that fire ants exploit resources by disrupting the mutualism between native ants and hemipteran insects. Under laboratory conditions, we have demonstrated that tending by both *S. invicta* and *T. melanocephalum* has positive impacts on the fitness of individuals but not small colonies of mealybugs. We did not find a difference between *S. invicta* and *T. melanocephalum* in terms of the benefit to mealybugs in the laboratory experiments. Compared with the absence of ant tending, lifespan was significantly extended and the number of offspring was increased when single mealybugs were tended by *S. invicta* or *T. melanocephalum* (Fig. 9). Honeydew removal and the stimulation of feeding by ants may be the primary reason for the observed mealybug colony growth. These results are consistent with previous studies indicating that ant tending was beneficial to the fitness of hemipteran colonies [6], [9], [20], [21], [64]. However, the benefit to mealybugs in the fire ant inclusion plot was greater than in the exclusion plot (where mealybugs were tended by native ants) indicated that fire ants facilitate mealybugs more than native ants. This may result from better protection provided by *S. invicta* than by native ants (Fig. 5, 6). Most importantly, our results suggested that foraging activity of workers of *T. melanocephalum* was more intensive than the activity of *S. invicta* workers when the two ant species were separated. Workers of *T. melanocephalum* discovered honeydew and recruited nestmates more quickly than workers of *S. invicta*. However, foraging intensity of *T. melanocephalum* was restrained by interference from *S. invicta* when the two ant species were foraging on one plant. We assigned equal biomass to the two ant species in our experiments. For a given biomass, there were more workers of *T. melanocephalum* than of *S. invicta*, which may contribute to the more intense foraging activity of *T. melanocephalum* in the first two observations. Food preference and olfaction difference are likely other reasons for this result. Workers of *T. melanocephalum* seemed to be more interested in honeydew than workers of *S. invicta*. However, with an increasing number of foraging workers of *S. invicta*, the advantage of greater foraging intensity of *T. melanocephalum* gradually disappeared (Fig. 13). Workers of *T. melanocephalum* were not completely driven off the plants by *S. invicta* in our experiment. Head-on confrontation happened occasionally between the two ant species. Workers of *T. melanocephalum* initially kept away from *S. invicta* most of the time. The results indicated that domination of food resources by *S. invicta* depended on their forceful aggressiveness. Colonies of *T. melanocephalum* were unlikely to take possession of honeydew when the numbers of foraging workers was nearly equal to that of *S. invicta*.

In conclusion, the mutualism between *S. invicta* and *P. solenopsis* facilitates population increase and fitness of each other. Although native ants may also establish mutualistic relationships with *P. solenopsis*, *S. invicta* tends to acquire more honeydew and therefore play a predominant role. Compared with native ants, *S. invicta* acquires most of the honeydew and protects *P. solenopsis* more effectively, which may facilitate the invasion of these two alien species in South China. While such mutual invasion success is mainly due to the aggressive behavior of fire ants which have further inhibition effect on native ants. Our results support a facilitative relationship of invasion between two exotic and mutualistic species. In addition, we should pay more attention to the invasion success facilitated by native species on *S. invicta* and *P. solenopsis* because their interactions with native species occur extensively when these alien species are introduced independently to a new area. Exploring these interactions with native species will help to further explain the invasion success of *S. invicta* and *P. solenopsis*.
